# Integrative interaction of emotional speech in audio-visual modality

**DOI:** 10.3389/fnins.2022.797277

**Published:** 2022-11-11

**Authors:** Haibin Dong, Na Li, Lingzhong Fan, Jianguo Wei, Junhai Xu

**Affiliations:** ^1^Tianjin Key Lab of Cognitive Computing and Application, College of Intelligence and Computing, Tianjin University, Tianjin, China; ^2^Brainnetome Center, Institute of Automation, Chinese Academy of Sciences, Beijing, China

**Keywords:** audio-visual integration, emotional speech, fMRI, left insula, weighted RSA

## Abstract

Emotional clues are always expressed in many ways in our daily life, and the emotional information we receive is often represented by multiple modalities. Successful social interactions require a combination of multisensory cues to accurately determine the emotion of others. The integration mechanism of multimodal emotional information has been widely investigated. Different brain activity measurement methods were used to determine the location of brain regions involved in the audio-visual integration of emotional information, mainly in the bilateral superior temporal regions. However, the methods adopted in these studies are relatively simple, and the materials of the study rarely contain speech information. The integration mechanism of emotional speech in the human brain still needs further examinations. In this paper, a functional magnetic resonance imaging (fMRI) study was conducted using event-related design to explore the audio-visual integration mechanism of emotional speech in the human brain by using dynamic facial expressions and emotional speech to express emotions of different valences. Representational similarity analysis (RSA) based on regions of interest (ROIs), whole brain searchlight analysis, modality conjunction analysis and supra-additive analysis were used to analyze and verify the role of relevant brain regions. Meanwhile, a weighted RSA method was used to evaluate the contributions of each candidate model in the best fitted model of ROIs. The results showed that only the left insula was detected by all methods, suggesting that the left insula played an important role in the audio-visual integration of emotional speech. Whole brain searchlight analysis, modality conjunction analysis and supra-additive analysis together revealed that the bilateral middle temporal gyrus (MTG), right inferior parietal lobule and bilateral precuneus might be involved in the audio-visual integration of emotional speech from other aspects.

## Introduction

Our senses enable us to receive information about our surroundings, and they also have the ability to perceive specific modalities. Sometimes we may be able to make accurate judgments and responses to the surroundings through information from only one modality, but more often we need the assistance of the integrated multi-sensory information, and our cognition of the world is often multimodal. Therefore, our sensory organs are not isolated from each other. It is their synergy that enables us to respond flexibly to changes in our environment, which depends on the integration of multimodal information by the human brain. As an important dimension of daily communication, emotion is expressed in multimodal ways, such as facial expressions, body movements, emotional speech and music. Studies have shown that when emotions are expressed consistently in visual and auditory modalities, the classification accuracy of emotion is higher than that of unimodal stimuli (Collignon et al., 2008; Hagan et al., 2013). Lots of researches have been carried out to reveal the integration mechanism of emotional information in visual and auditory modalities. These studies mainly focus on the integration of static or dynamic facial expressions and emotional rhythm or music, and rarely involve the integration of dynamic facial expressions and emotional speech. Consistent visual and musical stimuli have been shown to be more capable of arousing strong emotional perception, and audio-visual integration can be triggered by emotional music, no matter what valence is. For music with positive emotions, the integration effect is more obvious. For music with negative emotions, the duration of visual information presentation has a more significant regulating effect on emotions (Baumgartner et al., 2006; Pan et al., 2019). Compared with the emotional rhythm or music, emotional speech is closer to daily communication, and the appearance of cross-modal integration in emotional speech is known. It is meaningful and feasible to explore the representation mechanism of emotional speeches (McGurk and MacDonald, 1976).

Visual and auditory emotional information have been adopted as stimulus materials in many studies, revealing the role of the superior temporal sulcus (STS) and superior temporal gyrus in integrating audio-visual emotional contents (Kreifelts et al., 2007; Robins et al., 2009; Park et al., 2010; Müller et al., 2012). Using positron emission tomography (PET), researchers have explored the brain regions activated when perceiving a combination of facial expressions and emotional sounds, and found that bimodal stimuli activated the left posterior temporal cortex more than unimodal stimuli (Pourtois et al., 2005). Another functional magnetic resonance imaging (fMRI) study using consistent non-verbal audio-visual emotional stimuli showed that the sound sensitive areas and facial sensitive areas were located in different areas of the STS, and the audio-visual integration area of emotional information was represented in STS where the sound and facial sensitive areas overlapped (Kreifelts et al., 2009). Another study explored the audio-visual integration mechanism of emotional speech with magnetoencephalography, and suggested the role of right STS in audio-visual integration of emotional speech (Hagan et al., 2013). The findings of these studies have pointed the audio-visual integration sites of emotional contents to STS. The electrophysiological experiments on monkeys and fMRI studies in humans have shown that the STS plays an important role in facial processing, also in human cognition including language, audio-visual integration and motion perception (Iidaka, 2012). A number of studies have explored the functionality of STS in greater depth. Bilateral superior temporal region has been suggested to play different roles in emotion perception and audio-visual integration. The effect of emotion perception is more obvious in bilateral superior temporal gyrus (STG), and the effect of audio-visual integration is distributed in bilateral posterior superior temporal sulcus (pSTS) and right anterior STS/gyrus. Furthermore, the role of pSTS in audio-visual integration has also been recognized by more studies (Müller et al., 2012; Watson et al., 2014; Straube et al., 2018).

A study on audio-visual language processing using EEG has shown that STS is neither the earliest nor the most significant activated site, although it is often considered to be necessary for the integration of audio-visual language (Bernstein et al., 2008). The role of other brain regions in audio-visual integration was also reported in a number of studies. Functional imaging studies using blood oxygen level dependence signal changes to observe the cross-modal integration of non-verbal audio-visual stimuli found that the region of cross-modal interaction was the most significant in the superior thalamus, including the STS, the inner parietal sulcus, the insula and part of the frontal lobe (Calvert et al., 2001). By exploring the brain regions involved in speech signal processing, one PET study revealed the role of pSTS in the interaction of cross-modal speech, and the supra-additive response of the right postcentral gyrus (Kang et al., 2006). Another study on non-verbal emotional information observed that the activation of bilateral posterior STG and right thalamus were enhanced by the audio-visual condition vs. auditory or visual condition (Kreifelts et al., 2007). And neurons in the ventral prefrontal cortex could be capable of integrating facial and vocal stimuli, the region of which is considered to be crucial for processing, integrating and remembering the stimuli of face and voice. These findings are also applicable to non-human primates (Diehl, 2012; Romanski, 2017). Although a large number of studies have confirmed the role of STS or part of it in audio-visual affective integration, a considerable number of studies have revealed that other brain regions might also be involved in audio-visual integration. One drawback is that these studies rarely involved emotional speeches. The controversy persists about how audio-visual emotional speech information is processed by our human brain and which brain regions are involved in audio-visual integration. More reliable brain networks may be revealed by the application of new methods for multifaceted analysis.

Most studies on audio-visual integration of emotional information used the traditional generalized linear model (GLM) to model the data, and reported the significantly activated voxels by the statistical analysis, such as the modality conjunction analysis and supra-additive analysis. Modality conjunction analysis is defined as the activation of visual and auditory stimuli (AV) stronger than the activation of any unimodal stimuli (AV > A)∩(AV > V). It can be used to locate brain regions that are specific to both auditory and visual stimuli, which may be associated with cross-modal emotional processing, such as the audio-visual integration of emotion. The supra-additive analysis is defined as that the activation intensity of AV is greater than the sum of two unimodality (A+V) which uses a single-cell record directly and is first defined and measured at the cellular level. The supra-additive analysis is also a valid method for obtaining the brain regions of cross-modal integration. It is relatively simple to process data using the modality conjunction analysis and supra-additive analysis, and the fine-grained pattern information can be lost by using the statistical analysis to report significantly activated voxels (Haynes and Rees, 2006; Norman et al., 2006). As a computational method of multivariate response pattern analysis, representational similarity analysis (RSA) besides these two traditional methods were introduced to analyze the activation pattern information in the whole brain (Kriegeskorte et al., 2008). In addition, we also used a weighted RSA method to analyze the contribution of each candidate model in the best fitted model, to further explain the representation of ROIs. New findings may be obtained by applying new methods, and more convincing brain regions may be obtained by taking the results of various analyses into consideration. Based on the results of previous studies, we hypothesize that some regions of the insula and temporal lobes may be involved in audio-visual integration of emotional speech processing.

In this study, subjects experienced four emotional stimuli (anger, sadness, neutral and joy) expressed by three modalities (visual, auditory and audio-visual) to explore the brain regions involved in the audio-visual integration of different emotional valences. GLM was first used to obtain the beta files corresponding to each stimulus condition. Next, the ROIs were determined by using the activation of all the subjects under the condition of audio-visual stimuli, and the candidate models were constructed by using hypothesis-driven RSA. The beta file was then generated using RSA based on ROI to obtain the correlation between the neural representation of ROIs and the candidate models. Weighted RSA was used to calculate the weight of each candidate model in the best fitted model for ROIs, thus obtaining the contributions of the models to interpret the representation of ROIs. To obtain brain regions that may be involved in audio-visual integration in the whole brain, a whole-brain searchlight method was then conducted to explore the brain regions significantly related to the audio-visual model, which could be used to verify the results of ROIs and locate other possible brain regions (Kriegeskorte et al., 2006). Finally, modality conjunction analysis and supra-additive analysis were conducted to further determine the potential regions involved in the audio-visual integration of emotional speech processing.

## Materials and methods

### Participants and experiment stimuli

Twenty-five healthy volunteers were recruited (ten females mean age 23.3 ± 1.40 years, range from 21 to 26 years) from Tianjin University in this study. Nine subjects were excluded for further analysis for the data quality control. All subjects were right-handed with normal or corrected-to-normal vision, and all subjects had no history of neurological and psychiatric disorders. The study was carried out in accordance with the recommendations of Institutional Review Board (IRB) of Tianjin Key Laboratory of Cognitive Computing and Application, Tianjin University. All subjects gave written informed consents in accordance with the Declaration of Helsinki.

**FIGURE 1:**
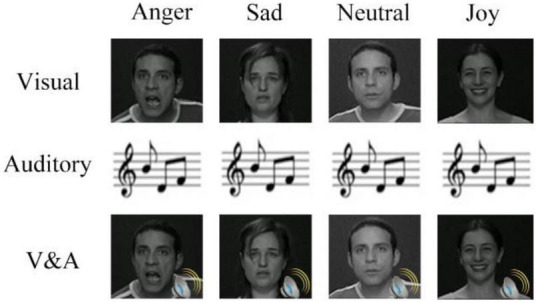
Exemplar stimuli used in the study. Four emotions and three modalities were included. Visual or auditory stimuli consisted of dynamic facial expressions or short sentences of emotional speech for 2 s. Audio-visual stimuli presented the visual and auditory stimuli at the same time.

The stimuli used in this study were collected from Geneva Multimodal Emotion Portrayals (GEMEP) (Bänziger et al., 2012). Four emotional videos were adopted in our experiment, including anger, sad, neutral and joy. There were three modalities, which were visual, auditory and audio-visual. The original materials were processed to dynamic facial expressions, emotional speech and audio-visual stimuli, with a duration of 2 s. Figure 1 shows exemplar stimuli materials in the experiment.

### Procedure and data acquisition

The fMRI experiment used an event-related design and contained three runs. The first run was visual stimuli of dynamic facial expressions, the second run was AV of emotional speech and the last run was consistent audio-visual emotional stimuli (Xu et al., 2021). Each run had 40 trials, including 10 trails for each condition of anger, sad, neutral and joy. In each trial, the stimuli displayed for 2 s, and the inter stimulus interval was 4–6 s with an average interval of 5 s. In each run, the order of the stimuli was pseudo randomly.

The imaging data was collected at Tianjin Huanhu Hospital using a 3.0T Siemens magnetic resonance scanner and an eight-channel head coil with the parameters in Table 1.

**TABLE 1:** The detailed parameters of functional and anatomical images.

Images	TR (ms)	TE (ms)	Voxel size (mm^3^)	Slices	FA	FOV (mm^2^)
Functional	2,000	30	3.1 × 3.1 × 4.0	33	90°	200 × 200
Anatomical	1,900	2.52	1 × 1 × 1	56	9°	256 × 256

TR, repetition time; TE, echo time; FOV, field of view.

### Data analysis

#### Regions of interest definition

The peak activation coordinates were first obtained in the activation results under the audio-visual condition at the group level (*p* = 0.05, FWE corrected). Voxels with a radius of 18 mm within the coordinate were extracted to obtain the main activated brain regions. Then the main activated regions were multiplied with the corresponding region of the AAL template, and the regions that might be involved in audio-visual information processing were obtained finally.

Nine ROIs were finally defined, including the left fusiform, bilateral lingual gyrus, STG, bilateral insula and bilateral pSTS. Table 2 shows the details of the regions and their MNI coordinates.

**TABLE 2:** Information of main activated regions under the audio-visual condition.

Anatomical region	Hemisphere	MNI coordinates		
		x	y	z
Fusiform, Lingual	L	–42	–48	–18
Fusiform, Lingual	L	–21	–75	–9
Lingual	R	15	–81	–3
STG, Insula, pSTS	L	–54	–15	9
STG, Insula, pSTS	R	57	–15	12

STG, superior temporal gyrus; pSTS, posterior superior temporal sulcus; L, left; R, right.

#### Data processing

The fMRI data was first preprocessed using the SPM12 toolkit in MATLAB software, including slice timing, realign, coregister, segment, normalize and smooth. In addition, the head movement is acceptable, which the horizontal movement is less than 2 mm and the rotation angle is less than 1.5^°^. The GLM analysis was conducted for each subject after preprocessing. GLM is based on the assumption that the experimental data on each voxel is a linear combination of unknown parameters, which is represented by betas. In this study, the unknown parameters that we are interested in contain 12 stimulus conditions, including four emotions (anger, sad, neutral and joy) expressed by three modalities (visual, auditory, and audio-visual). The purpose of the GLM analysis is to calculate the beta values corresponding to each condition.

For each ROI, the beta values of all conditions were extracted and expanded to a matrix of 12 × n (n represents the number of voxels in the ROI). Then the traditional RSA was used to compute the representation dissimilarity matrices (RDMs) of ROIs. The RDMs could be obtained by calculating the dissimilarity values between each two conditions, which were defined as one minus Pearson correlation coefficients. Next, the candidate model RDMs were constructed using hypothesis-driven RSA. Five emotion models (anger, sad, neutral, joy, and negative) and three modality models (visual, auditory and audio-visual) were constructed. In the emotion models, the dissimilarity values between different modalities of the same emotion were set to 0 and the dissimilarity between other conditions was set to 1. The generation of modality models were similar to the emotion models.

In addition, a weighted RSA method was used to examine the contributions of all candidate models to the best fitted models of ROIs (Xu et al., 2021). The best fitted model of a ROI was obtained by using the weighted linear sum of the 8 candidate models to make the mean square error between the neural representation RDM of the ROI and the fitted model minimized. The weights of candidate models could be used to explain the representation of one region.

The statistical inference was finally made in the ROI analysis. For each ROI, the Kendal rank correlation coefficients between the neural representation RDM and the model RDMs were calculated. The participants were treated as random effects in the group analysis and the Kendall rank correlation coefficients were submitted to a unilateral Wilcoxon sign rank test to evaluate the significance of the correlation.

**FIGURE 2:**
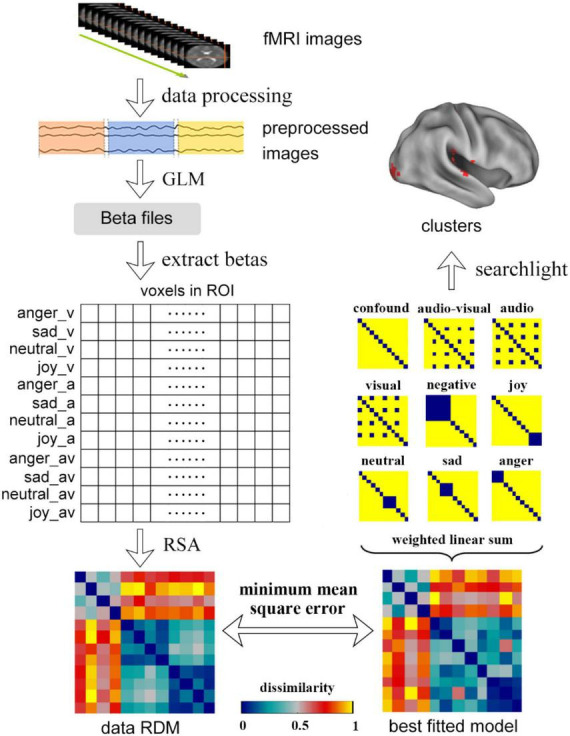
The procedure of data processing, including the process of traditional RSA, weighted RSA and searchlight. In the traditional RSA, the data was first preprocessed and was modeled by GLM to get the beta files. The betas in the ROIs were extracted and were used to calculate the data RDMs using RSA. Using weighted RSA, the weighted linear sum of all candidate models was used to obtain a best fitted model to minimize the mean square error between the data RDM and fitted model. The clusters that significantly correlated to a candidate model could be obtained by using whole brain searchlight analysis.

The searchlight analysis was used to obtain the regions that were significantly correlated to a specific candidate model in the whole brain. For each voxel, the beta values of the neighbor voxels with a radius of 6 mm were extracted and expanded to a matrix of 12 × n. Then the searchlight RDM could be obtained by using RSA. The correlation coefficient between data RDM and the candidate model was calculated, which was assigned to the central voxel of the searchlight analysis. The subjects’ whole-brain correlation map (r-Map) was obtained by repeating the process for all subjects on the whole brain. The subjects were treated as random effects in the group analysis and a one-sample *t*-test was used to get the significance value corresponding to the candidate model, then the whole-brain significance map (p-Map) was acquired. The whole procedure of data processing is shown in Figure 2.

### Modality conjunction analysis and supra-additive analysis

To further locate which brain region may be involved in audio-visual integration, two contrasts were defined in the first level analysis for all subjects. Then the group level analysis was conducted on all subjects’ result by using one sample *t*-test, and the significance level is set to *p*0.05. The activation map of audio-visual stimuli vs. unimodal stimuli was obtained on the group level. Finally, a conjunction analysis of (AV > A)∩(AV > V) was conducted.

Supra-additive analysis can be used to explore brain regions involved in processing other information besides visual and AV, such as audio-visual integration. The contrast of AV > (A+V) was set in the first level analysis for all subjects. The activation map was obtained by using one sample *t*-test with significance level of *p*0.05 for the group level.

## Results

### Neural representations in the weighted representational similarity analysis

Nine ROIs were defined using the activation map of emotional speech at a group level of all subjects under the condition of audio-visual stimuli. To explore whether these brain regions are truly involved in the audio-visual integration of emotions, it is necessary to examine the association between the representation of brain regions and the emotional models, and determine whether they are significantly correlated with the audio-visual model.

**FIGURE 3:**
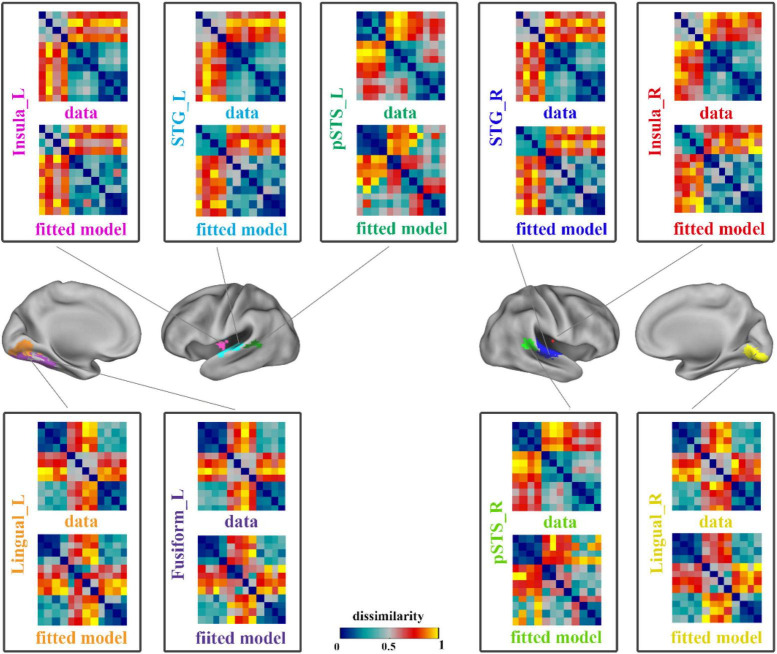
The results of data RDMs and best fitted model RDMs on ROIs. Each pane showed the neural representation (data) RDM and the fitted model RDM of a ROI. The four panes on the right showed the results of right hemisphere and others showed the left hemisphere’s results. STG, superior temporal gyrus; pSTS, posterior superior temporal sulcus; L, left; R, right.

A linear combination of the 8 candidate models that constructed in the RSA procedure based on ROIs was used to obtain the optimal fitted model corresponding to each region, so as to minimize the mean square error between the fitted model and the neural representation RDM. The neural representation RDMs and the best fitted model RDMs of all brain regions are shown in Figure 3.

Table 3 shows the weight information of the candidate models in the best fitted model of each region. Since our purpose is to reveal the integration mechanism of emotional speech in audio-visual modality, we are more interested in the emotion models and the audio-visual model. Only the weight values of four single emotional models and audio-visual modal model are shown.

**TABLE 3:** The weights of each model in fitted model (*p* = 0.05, FDR corrected).

Region	Anger		Sad		Neutral		Joy		Audio-visual	
	Mean	SD	Mean	SD	Mean	SD	Mean	SD	Mean	SD
Fusiform_L	0.262	0.225	0.003	0.038	0.016	0.026	0.051	0.105	0.027	0.016
Lingual_L	0.259	0.237	0.012	0.084	0.014	0.023	0.042	0.086	0.025	0.017
Lingual_R	0.286	0.296	0.001	0.002	0.005	0.016	0.103	0.174	0.016	0.017
STG_L	0.335	0.382	0.019	0.009	0.006	0.006	0.029	0.066	0.019	0.017
STG_R	0.328	0.368	0.028	0.025	0.002	0.002	0.034	0.030	0.028	0.014
Insula_L	0.206	0.287	0.089	0.122	0.012	0.068	0.086	0.141	0.039	0.020
Insula_R	0.276	0.334	0.040	0.037	0.008	0.008	0.086	0.143	0.027	0.005
pSTS_L	0.109	0.196	0	0	0.015	0.009	0.097	0.054	0	0
pSTS_R	0.168	0.240	0	0	0.004	0.003	0.073	0.052	0	0

SD, standard deviation; STG, superior temporal gyrus; pSTS, posterior superior temporal sulcus; L, left; R, right.

The weight analysis showed that the weights of the sad model and audio-visual model were zero in the bilateral pSTS, and the weights of other emotion models were also not so high, suggesting that the bilateral pSTS might not be involved in the integration of audio-visual emotional speech. For the results of the best fitted models in other ROIs, each of the candidate models showed a great contribution, indicating that they might be associated with the audio-visual integration of emotions. It is worth noting that, compared with other brain regions, the audio-visual model had a more significant contribution to the best fitted model of the left insula (Mean = 0.039, *SD* = 0.020), indicating that the left insula was more likely to participate in the audio-visual integration of emotional speeches among all ROIs.

### Statistical analysis on the neural representation of regions of interests

**FIGURE 4:**
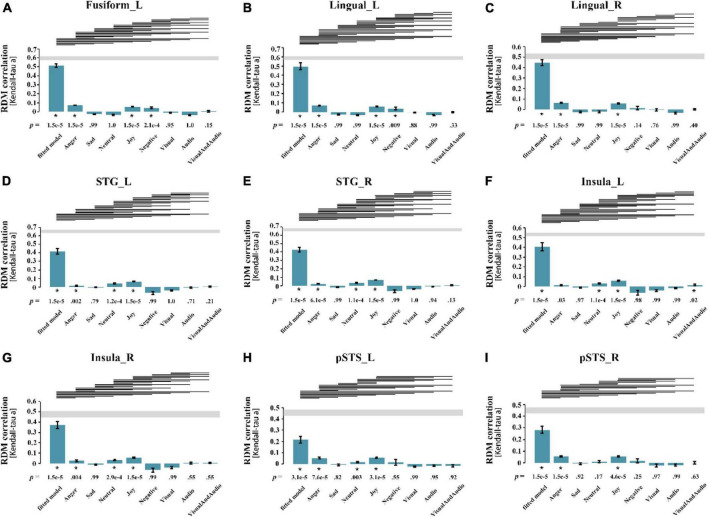
Results of statistical analysis for all ROIs. Each pane corresponded to the result of a ROI, the abscissa represents the best fitted model and 8 candidate models from left to right, and the ordinate represents the Kendall correlation coefficient between the neural representation RDM of the brain region and each model. **(A)** left Fusiform gyrus; **(B)** left Lingual gyrus; **(C)** right Lingual gyrus; **(D)** left superior temporal gyrus; **(E)** right superior temporal gyrus; **(F)** left Insula; **(G)** right Insula; **(H)** left posterior superior temporal sulcus; **(I)** right posterior superior temporal sulcus. The *p*-value at the bottom of the bar represents the significance, and the asterisk represents the correlation between neural representation RDM and the model is significant (*p* = 0.05, FDR corrected). The line segment at the top of the panel indicates that the performance of the two models is significantly different, and if the model on the left performs better than the one on the right, there is a line segment from left to right. STG, superior temporal gyrus; pSTS, posterior superior temporal sulcus; L, left; R, right.

For each subject, the Kendall rank correlation coefficients between the neural representation RDMs of the ROIs and the candidate models were further calculated. In the group analysis, the unilateral sign rank test (*p* = 0.05, FDR corrected) was used to process the results of all subjects, in order to obtain the correlation and significance between the RDMs and the models. Figure 4 shows the result of the statistical analysis, indicating that for all ROIs, only the left insula shows a significant correlation with the audio-visual model, further confirming that the left insula may be involved in audio-visual integration of emotional speech.

Since sadness is greatly affected by the individual differences, the representation of sadness is not considered here. The statistical analysis shows that the neural representation RDMs of the bilateral STG, right insula, and left STS showed significant correlations with all emotion models except the sad model, suggesting that they may be involved in the processing of emotional information, although their correlations with the audio-visual model are not significant. The statistical analysis of RSA should not be the only criterion for evaluation. And whether these brain regions are involved in audio-visual integration of emotional speech needs further exploration. The neural representation RDMs in the left fusiform gyrus, bilateral lingual gyrus and right pSTS were not significantly correlated with the sad model and neutral model, suggesting that they may not be involved in the processing of neutral emotions, and therefore may not be associated with the audio-visual integration of emotions in different valences.

Moreover, the best fitted models obtained by weighted RSA are significantly better than other candidate models, which further demonstrates the superiority and reliability of weighted RSA.

### Searchlight analysis

In this study, only the left insula was found to be involved in audio-visual integration of emotional speech by the RSA method. To further verify whether the left insula and other ROIs were involved in audio-visual information processing and locate other brain regions that might be involved in audio-visual integration, a searchlight analysis was conducted to calculate voxels that were significantly correlated with the audio-visual model in the whole brain, with a radius of 6 mm. One sample *t*-test was performed using *p*-value of 0.05 with a cluster size of not less than 30 voxels to locate all regions that might be associated with audio-visual integration. Table 4 shows the peak coordinates of the clusters that were significantly correlated to the audio-visual model.

**TABLE 4:** The searchlight results of audio-visual model (*p* = 0.05, one sample *t*-test).

Anatomical region	Hemisphere	MNI coordinates			Cluster size
		*x*	*y*	*z*	
Insula	L	–36	–15	–6	77
Temporal_Sup_R (aal)	R	63	3	–3	60
Lingual_L (aal)	L	–15	–81	–18	56
Fusiform_L (aal)	L	–42	–60	–27	46
Temporal_Mid_L (aal)	L	–15	–102	–12	39
Temporal_Mid_R (aal)	R	54	–24	–21	33

The cluster size indicated number of voxels; L, left; R, right.

The searchlight analysis shows that the representation of the left insula is significantly associated with the audio-visual model, further confirming its crucial role in the audio-visual integration of emotional speech. The right STG is also observed to be involved in audio-visual integration. In addition, some other regions are identified, including the bilateral middle temporal gyrus (MTG). Further analysis will be carried out to reveal whether audio-visual integration of emotional speech is indeed associated with these brain regions.

### Modality conjunction analysis and supra-additive analysis

To further determine which observed regions are really involved in the audio-visual integration of emotional speech, the modality conjunction analysis of (AV > A)∩(AV > V) and the supra-additive analysis of (AV > A+V) were conducted for all subjects.

**FIGURE 5:**
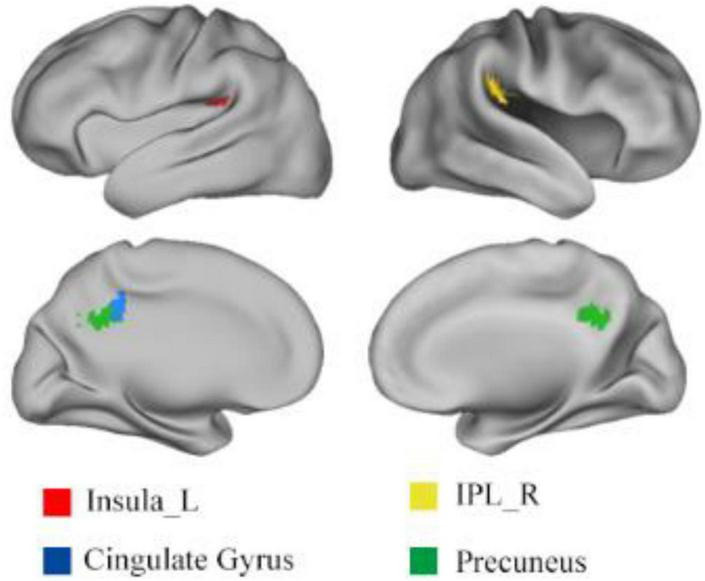
Results of (AV > A) ∩ (AV > V). IPL, inferior parietal lobule; L, left; R, right.

Using one sample *t*-test of *p* = 0.05 and cluster size above 30 voxels, the modality conjunction analysis (Figure 5) reveals that the left insula, right IPL, bilateral precuneus and bilateral cingulate gyrus show more enhanced activations in audio-visual condition than any unimodal stimuli of visual or auditory. The regions that were more activated by audio-visual stimuli vs. the sum of auditory and visual stimuli were further obtained, as shown in Table 5. Meanwhile, the supra-additive analysis found that the activations of bilateral IPL, bilateral precuneus, bilateral MTG and left insula by audio-visual stimuli were greater than that of the sum of visual and AV.

**TABLE 5:** The results of (AV > A + V) (*p* = 0.05, one sample *t*-test).

Anatomical region	Hemisphere	MNI coordinates			Cluster size
		*x*	*y*	*z*	
Inferior parietal lobule	R	48	–75	21	228
Precuneus_R (aal)	R	9	–48	3	225
Precuneus_L (aal)	L	9	–48	3	195
Inferior parietal lobule	L	–45	–81	18	182
Temporal_Mid_L (aal)	L	–57	–24	–18	48
Middle temporal gyrus	R	48	–75	21	47
Insula	L	–42	–21	0	43

The cluster size indicated number of voxels; L, left; R, right.

The RSA based on ROIs indicates that only the left insula shows a significant correlation with the audio-visual model, and the audio-visual model shares a higher weight in the best fitted model of the left insula compared with other brain regions. The whole-brain searchlight analysis also found the left insula was significantly correlated to the audio-visual model. The modality conjunction analysis and supra-additive analysis reveal that the activation of the left insula in audio-visual stimuli is greater than either unimodal stimuli or the sum of them. Only the left insula is detected by all methods among the ROIs, indicating that the left insula plays an irreplaceable role in the audio-visual integration of emotional speech.

It fails to detect other brain regions that were significantly associated with the audio-visual model except the left insula. However, our findings show that neural representation RDMs of the bilateral STG, right insula and left pSTS have significant correlations with all emotion models except the sad model, suggesting that these brain regions may be involved in multimodal processing of emotional information. Other ROIs are excluded from the regions involved in audio-visual integration of different valence emotions because they fail to show significant correlations with the neutral emotion model. Further searchlight analysis suggests that the right STG is significantly correlated with the audio-visual model, and bilateral MTG may be involved in audio-visual information processing. However, neither the modality conjunction analysis nor supra-additive analysis observed that the right STG was associated with audio-visual integration, indicating that it may only receive audio-visual emotional information, but is not involve in the integration of it.

The bilateral MTG was found in the supra-additivity. Considering the finding of the modality conjunction analysis and supra-additive analysis, the right IPL and bilateral precuneus may also be involved in the processing of audio-visual information. Although these brain regions are not included in the analysis of ROIs, the searchlight analysis, modality conjunction analysis and supra-additive analysis reveal that the bilateral MTG, right IPL and bilateral precuneus may be involved in the audio-visual integration of emotional speech from other aspects.

## Discussion

This study conducted the traditional RSA, weighted RSA, whole-brain searchlight analysis, modality conjunction analysis, and supra-additive analysis to explore the brain regions that may be involved in the audio-visual integration of different emotional valences. The left insula, which is detected by all methods, is found to play a crucial role in the audio-visual integration of emotional speech. The whole-brain searchlight analysis and supra-additive analysis reveal that the bilateral MTG may also be involved in audio-visual processing of emotional speech. The right IPL and bilateral precuneus are also observed in the processing of audio-visual information using the modality conjunction analysis and supra-additive analysis. All these findings suggest that the left insula, bilateral MTG, right IPL and bilateral precuneus may constitute a specific brain network and participate in the integration of multimodal emotional information.

### The integration mechanism of emotional speech in the audio-visual modality

The main finding of this study is that only the left insula is significantly correlated with the audio-visual model. Weighted RSA shows that the weight of the audio-visual model in the best fitted model of the left insula was greater than that of other brain regions. The left insula is also observed in the whole-brain searchlight analysis. Modality conjunction analysis and supra-additive analysis indicate an enhanced activation in the left insula on the condition of audio-visual stimuli compared with unimodal stimuli and the sum of visual and AV. All these findings suggest that the left insula is engaged in the processing of emotional speech in audio-visual modality and plays a central role in the specific emotion processing network. The insula has long been considered as an important processing center to the cognitive processing, including emotional processing, somatic movement and working memory, and it plays an important role in multimodal sensory processing, audio-visual integration and emotion (Xu et al., 2019). The insula has been confirmed to be capable of evoking relevant emotions, thus forming a conscious perception of emotion (Schachter and Singer, 1962; Russell, 2003). One study using the combination of negative smell and disgust expressions found that the signals of the anterior insula were regulated, which indicated that the insula may participate in the integration of different sensory signals (Seubert et al., 2010). Another study also suggested that consistent emotional stimuli of facial and vocal could trigger the activation of insula, which provided evidence to the participation of the insula in the integration of audio-visual emotional information (Klasen et al., 2011). Our study found that the neural representation in left insula was significantly correlated with audio-visual models, suggesting that in the case of cross-modal stimuli, the left insula may participate in receiving the information and then conduct further processing in cooperation with other brain regions. The activation of the left insula is greater in the audio-visual condition than that of any unimodal stimuli, and the supra-additivity is also observed in insula, revealing its role in integrating audio-visual information. Evidence from the studies of electrophysiological and the process of hemispheric inactivation indicates that the internal emotional processing is strongly left-biased based on the autonomous input of insula (Oppenheimer et al., 1992). Strong similarities were found with previous studies on the function of insula and provide further evidence for the involvement of the left insula in the reception and integration of audio-visual emotional speech.

Parts of bilateral MTG were found to be significantly correlated with the audio-visual model in our whole-brain searchlight analysis. The modality conjunction analysis suggests that the activations of the right IPL and bilateral precuneus were stronger on the condition of audio-visual emotional stimuli than that of any unimodal stimuli. Furthermore, supra-additivity is detected in the right IPL, bilateral precuneus and bilateral MTG. These results together indicate that these brain regions may be involved in the audio-visual integration of emotional speech from other aspects.

One previous study that used the auditory or visual features of animals or artificial tools to perform recognition tasks found that the MTG had enhanced BOLD response compared to unimodal stimuli, when auditory and visual stimuli were presented simultaneously (Beauchamp et al., 2004). Another latest study have pointed out that the STG and MTG directly participate in the comprehensive audio-visual processing (Suh et al., 2019). And this study obtained one specific network processing the audio-visual information including the bilateral MTG. In addition, supra-additivity response is also found in bilateral MTG, suggesting that they may also be involved in the process of audio-visual integration. However, bilateral STG are not observed to be involved in the modality conjunction analysis, suggesting that they may only play an auxiliary role in the audio-visual integration of emotional speech. One relevant study has shown that the MTG plays an auxiliary but not necessary role in cross-modal integration, which is consistent with this study (Taylor et al., 2009).

The IPL has been found to be involved in multimodal integration of visual cues and head movements, suggesting its important role in multisensory integration (Schindler and Bartels, 2018). And previous studies have shown that the inferior parietal cortex is a brain region with multimodal functional heterogeneity, and the inner parietal lobe sulcus is associated with cross-modal interactions of non-verbal audio-visual stimuli (Calvert, 2001; Mueller et al., 2013). Our study shows that the right IPL is detected by the modality conjunction analysis and supra-additive analysis, indicating that the right IPL may also be involved in the audio-visual integration process of emotional speech.

The precuneus plays an important role in a series of highly integrated tasks, including visuo-spatial imagery, episodic memory retrieval and self-processing operations (Cavanna and Trimble, 2006). It not only plays a leading role in the default neural network, but also play a broader role in the multimodal processing tasks (Utevsky et al., 2014). The precuneus has been found to be significantly activated when performing motor and counting tasks, but its function connectivity pattern is different from that of other brain regions. This suggests that the precuneus is capable of monitoring the operations of other regions in the brain network (Wu et al., 2013). The precuneus has been activated in some studies on multitasking, and may not be directly involved in the processing of these tasks, but plays a supervisory and controlling role in the execution of these tasks. The finding of this study shows that the precuneus may indeed be involved in the integration process of audio-visual emotional speech by the modality conjunction analysis of (AV > A)∩(AV > V) and supra-additive analysis of (AV > A+V). However, the searchlight analysis fails to detect the significant correlation between the neural representation of precuneus and audio-visual model. It’s speculated that the audio-visual emotional speech recognition is a complex multitask process, which needs a specific brain network to complete. The precuneus does not actually participate in the processing of the task, but coordinates and controls the operations among other regions, ensuring the normal operation of the brain network. Furthermore, our study found that the audio-visual integration of emotional speech required the coordination of a brain network including the left insula, bilateral MTG, right IPL and bilateral precuneus. This specific network plays an auxiliary role in the integration of audio-visual information, in which the bilateral precuneus are not directly involved in the task but play a monitoring role during this cooperation.

### Regions that couldn’t integrate audio-visual emotional speech

The weighted RSA shows that the weights of the sad model and audio-visual model are both zero in the best fitted model of bilateral pSTS. Moreover, the neural representation RDMS of the left fusiform gyrus, bilateral lingual gyrus and right pSTS are not significantly correlated with the sad model and neutral model. In addition, none of the ROIs was observed in the modality conjunction analysis and supra-additive analysis except the left insula, suggesting that these brain regions may not be involved in the integration of multimodal emotional speech in different valences.

Previous studies have shown that the fusiform gyrus is specialized in processing facial information and is a typical facial response region (Haxby et al., 2000; Herrington et al., 2011). And it’s been suggested that the fusiform gyrus is involved in emotional perception and is related to the recognition of static facial expressions (Kesler et al., 2001; Bae et al., 2019). Lingual gyrus has long been thought to have two main functions: face processing and word processing. One recent study has pointed out that the lingual gyrus is associated with facial expression recognition, indicating that it not only participates in facial information processing, but also is involved in the processing of emotion (Kitada et al., 2010). Another study found that dyslexic children had less activations of the left lingual gyrus during alphabet processing tasks than normal children, further demonstrating the role of the lingua gyrus in word processing (Baillieux et al., 2009). In this study, the left fusiform gyrus and bilateral lingual gyrus have been found to be activated by the emotional speech and dynamic facial expression, but we don’t observe them participating in the audio-visual integration process in a series of subsequent analyses. This suggests that the activation of the left fusiform gyrus may be triggered by facial expression stimuli. The stimuli of emotional speech phrases and facial expression may induce the processing of these information by bilateral lingual gyrus, but they may not participate in the further processing of audio-visual emotion. Our study further reveals the role of the fusiform gyrus and lingual gyrus in facial emotion processing, which is consistent with previous studies.

A large number of studies on cross-modal audio-visual emotional interaction have shown that the STG and STS play a key role in integrating and controlling audio-visual emotional information (Kreifelts et al., 2007; Robins et al., 2009; Park et al., 2010; Müller et al., 2012; Hagan et al., 2013). However, it has also been suggested that the role of the STG and STS is unessential in multisensory integration. By dividing facial expressions into upper and lower parts to explore the integration mechanism of consistent or inconsistent facial emotional expressions, one latest study found that the fusiform gyrus and amygdala were the hub of the brain network, while STS and prefrontal lobe were more likely to conduct a partial analysis during the process of consistent emotions (Meaux and Vuilleumier, 2016). This indicated that STS was inadequate in its ability to integrate unimodal local information. The pSTS is also revealed to participate in cross-modal integration, but its role was not necessary (Taylor et al., 2009). Another study found that STS was neither the earliest nor the most significant activation region of audio-visual speech stimuli (Bernstein et al., 2008). Many studies have revealed some other regions that may be involved in emotional audio-visual integration, including amygdala, frontal lobe and thalamus (Calvert et al., 2001; Kreifelts et al., 2007; Domínguez-Borràs et al., 2019). Thus, it can be seen that the exact location of the brain region involved in cross-modal integration may be different due to the experimental paradigms, stimulus materials and other factors, and the involvement of the superior temporal region in audio-visual integration is not absolute. In this study, emotional speech is used to explore the mechanism of audio-visual integration, and the result fails to show that the STG and STS are involved in the integration process. However, these two regions have shown peak activations on the audio-visual condition, which is consistent with the finding of another study using audio-visual speech stimuli (Bernstein et al., 2008).

### Speech dual path model

The dual path model of speech has a ventral pathway and a dorsal pathway, the ventral pathway is called the “what” pathway, and the dorsal pathway is called the “where” pathway. The ventral pathway is mainly responsible for the mapping between encoding-meaning representations. The ventral pathway is mainly responsible for auditory information processing, and the neural network involved is mainly from the mid-anterior temporal lobe to the lateral ventral frontal lobe. Through the whole-brain searchlight analysis and supra-additive analysis, we found that the bilateral MTG is involved in the ventral pathway. The dorsal pathway is primarily responsible for the mapping between sensory-motor encodings. The dorsal pathway is mainly responsible for spatial information processing, and the neural network involved is mainly from the occipital visual cortex to the dorsal parietal pathway. Through the whole-brain searchlight analysis, the modality conjunction analysis and the supra-additive analysis, we found that the right IPL is involved in the dorsal pathway.

## Limitation

There are several issues that should be addressed in this study. Although a brain network involved in the audio-visual integration of emotional speech is identified in this study, including the left insula, right IPL, bilateral MTG and bilateral precuneus, the relationships among these brain regions and the process to achieve the integration are not clear, which is only be explained simply according to the results of RSA, modality conjunction analysis and supra-additive analysis, and how the brain network works remains to be explored. In future studies, the functional connectivity and effective connectivity can be considered to provide further evidence. Moreover, the number of participants recruited in this study is relatively less, and more convinced results can be obtained by increasing the quantity of subjects. In addition, the emotional stimuli used in the study are the same valence in the audio-visual modality. Different emotions expressed by different modalities can be considered to explore the integration mechanism of inconsistent audio-visual emotions in the future studies.

## Conclusion

In this study, the RSA based on ROIs, weighted RSA, whole-brain searchlight analysis, modality conjunction analysis and supra-additive analysis are used to explore the brain regions involved in audio-visual emotional integration of various valences. The results show that the audio-visual processing of emotional speech is conducted by a specific brain network, including the left insula, right IPL, bilateral MTG and bilateral precuneus. These brain regions are responsible for information reception and integration, information integration, assistance, supervision and coordination in the whole process. The findings show that our method can give insights into the research of cognitive neuroscience.

## Data availability statement

The original contributions presented in this study are included in the article/supplementary material, further inquiries can be directed to the corresponding author.

## Ethics statement

This study was reviewed and approved by Institutional Review Board (IRB) of Tianjin Key Laboratory of Cognitive Computing and Application, Tianjin University. Written informed consent was obtained from all participants for their participation in this study.

## Author contributions

JX proposed the idea. JX and NL designed the experiments. HD and NL performed the experiments and wrote the manuscript. JW, LF, and JX contributed to the manuscript revision. All authors contributed to discuss the results and have approved the final manuscript.
